# Emergency medicine physician burnout before and during the COVID-19 pandemic

**DOI:** 10.1186/s13584-022-00539-4

**Published:** 2022-08-24

**Authors:** Noaa Shopen, Assaf Schneider, Reut Aviv Mordechai, Malka Katz Shalhav, Efrat Zandberg, Moshe Sharist, Pinchas Halpern

**Affiliations:** 1 Department of Emergency Medicine, Tel Aviv Medical Center, Tel Aviv, Israel, affiliated to the Sackler Faculty of Medicine, Tel Aviv University, Tel Aviv, Israel; 2 Department of Emergency Medicine, Shamir Medical Center, Zerifin, Israel, affiliated to the Sackler Faculty of Medicine, Tel Aviv University, Tel Aviv, Israel

**Keywords:** Burnout, COVID-19, Pandemic, Emergency medicine physicians, Work overload, Work meaning

## Abstract

**Background:**

Burnout is a common issue among physicians, and the rate among emergency medicine physicians (EPs) appears to be higher than those of other medical specialties. The COVID-19 pandemic presents unprecedented challenges to the medical community worldwide, but its effects on EP burnout has not yet been determined.

**Methods:**

We conducted a three-stage nationwide study between July 2019 and June 2021. First, we evaluated the responses to burnout questionnaires that had been filled in by EP before the COVID-19 pandemic. We then re-sent the same questionnaires, with an addition of pandemic-specific questions. The third step involved a small group of EPs who participated in a burnout reduction workshop and re-took the questionnaires after a 3-month interval. The Maslach Burnout Inventory measured three burnout scales and a Work and Meaning Inventory predicts job satisfaction. Descriptive, univariate, and multivariate statistical tests were used to analyze the data.

**Results:**

In the first stage, 240 questionnaires were sent by email to all Israeli EPs listed in emergency departments nationwide, and 84 out of 88 submitted questionnaires were completed in full before the pandemic. 393 questionnaires were sent in the second stage during the pandemic and 93 out of 101 submitted questionnaires were completed in full. Twenty EPs participated in the workshop and 13 out of 20 submitted questionnaires were completed in full. Burnout levels were high (Maslach) among EPs before the pandemic and increased during the pandemic. The feelings of personal accomplishment and work meaning—both protective factors from burnout—were significantly higher in the second (pandemic) stage. The pandemic-specific burnout factors were fear of infecting family members, lack of care centers for the physician’s children, increased workload, and insufficient logistic support. The physician-oriented intervention had no significant impact on burnout levels (*p* < 0.412, Friedman test).

**Conclusions:**

Physician burnout is a major global problem, and it is now being aggravated by the challenges of the COVID-19 pandemic. Healthcare administrators should be alerted to pandemic-specific stress factors in order to help teams cope better and to prevent further worsening of the burnout. Further research is warranted to determine the lasting effect of the pandemic on EM physician burnout and the best means for reducing it.

## Introduction

Burnout is a common issue among physicians, especially EPs [1, 2]. It is associated with negative effects on quality of life, among them poorer mental and physical health, elevated blood pressure, sleep disturbances, and excessive use of non-prescription psychoactive substances and alcohol. [3–5] EPs worldwide appear to have a higher rate of burnout compared to most other medical specialties. [1, 6–9] Paradoxically, they also have high rates of work satisfaction. [8, 10, 11]. The COVID-19 pandemic is presenting unprecedented challenges to the global medical community, but data on its effect on physician burnout are still limited. A literature review of the impact of previous pandemics and natural disasters on the mental health of healthcare workers concluded that these events increase burnout and may cause post-traumatic stress disorder. [12]. Another review concluded that public health, EM, primary care, and intensive or critical care workers are particularly at high risk of developing psychological symptoms due to the pandemic. [13].

In this study, we continue the work done by our group in identifying and analyzing burnout among EPs [10]. Fortuitously, we sent a validated burnout and work satisfaction questionnaire to Israeli EPs, both residents and attending physicians, just prior to the pandemic. We exploited the availability of these data to continue the study and investigate pandemic-specific burnout factors and changes in EM physician burnout characteristics during the pandemic.

The objectives of the current work were: (1) to identify the levels of burnout, its causes and potential protective factors in physicians who regularly work in EDs in Israel; (2) to identify the effect of personal and professional coping mechanisms with the global epidemic in terms of burnout in Israeli EPs; (3) to assess the ability of a physician-oriented intervention to reduce burnout levels.

## Methods

The study was performed in three stages:

Stage 1: Conducted from July 2019 through March 2020, before and up to the COVID-19 outbreak. An anonymous burnout questionnaire (see below) was sent to 15 Israeli EDs in which a total of 240 EPs (residents and attending physicians) were working over 20 h a week.^1^

Stage 2: Conducted during the COVID-19 pandemic from December 2020 through April 2021. A second anonymous questionnaire was sent to all Israeli EM physicians. This four-part questionnaire included items on burnout that were identical to those in the first stage, as well as questions on various factors of concern unique to the pandemic situation. The pandemic-specific questions were based on both preliminary studies done in the first weeks of the pandemic and the authors’ clinical experience working in the COVID-19 wing of the ED. [13, 14] The four parts were:

1. Demographics and mood
2. The Maslach Burnout Inventory that measures the three burnout scales of emotional exhaustion, depersonalization, and personal accomplishment. [15, 16]
3. A Work and Meaning Inventory (WAMI), which is a unique predictor of job satisfaction, number of days reported absent from work, and life satisfaction. [17]
4. Questions aimed to identify pandemic-specific burnout factors (for the second study group), including an open-ended question requesting the participants to state what they think might help them better cope with the extra workload caused by the pandemic.

Stage 3: This part was conducted from February 2021 through June 2021. A subgroup of residents and attending EPs from the ED of a single medical center^2^ participated in a three-part burnout reduction workshop. This workshop included group and individual sessions and was aimed at providing various tools for strengthening mental resilience that had been used before on medical and nursing staffs in other Israeli hospitals (e.g., Sheba, B’nei Zion), sports teams, and for members of the Prime Minister’s office staff [18]. The participants were requested to fill in the burnout questionnaires a second time at the end of the intervention program and again three months later. The interventional part of the study was initially intended to be conducted in several medical centers. However, the first workshops were conducted in February 2021, which was the time of the third quarantine in which COVID-19 morbidity was still high and strict post-exposure isolation regulations were in place. The EDs therefore declined to risk further exposure of physicians to the venues of the workshops.

The data were analyzed with IBM SPSS statistics software version 28.0. (SPSS Inc. Chicago, IL, USA). The significance levels were set at 0.05. Data were presented as mean and standard deviation (SD) for continuous variables and as frequency and percentage for categorical variables. Chi-square tests and independent t-tests were performed to compare the two groups (i.e., before and since pandemic onset) for categorical and continuous variables, respectively. The Friedman test was used for the individual matching of workshop participants.

## Results

**Fig. 1:**
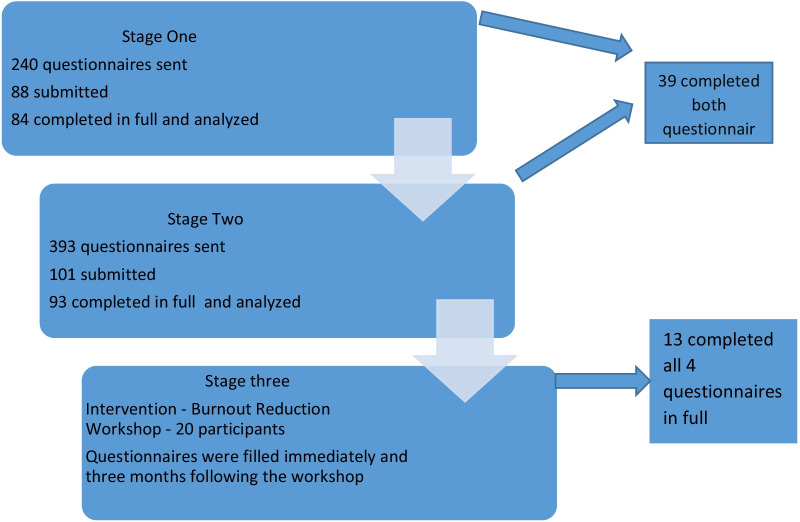
Flowchart of the survey

In the first stage, a total of 240 questionnaires were sent by email to Israeli EPs working in EDs (names and email addresses provided by the departments). The first stage was conducted before the COVID-19 pandemic, and 84 out of 88 submitted questionnaires were completed in full. The second stage was conducted during the COVID-19 pandemic, 393 questionnaires were sent and 93 out of 101 submitted questionnaires were completed in full. Thirty-nine EPs completed both questionnaires (Fig. 1).

As shown in Table 1, the two groups were similar in male-to-female ratio, marital status, number of children, and extent of physical activity. The second group had a slightly higher ratio of residents, and participants were therefore younger, had fewer years of ED work, and more 24 h shift.

**Table 1:** Participants’ demographics

	Before the pandemic	During the pandemic	*p*
	N = 88 (%)	N = 101 (%)	
*Age (years):*			0.039
< 40	45 (52)	66 (66)	
41–50	21 (24)	17 (17)	
> 51	21 (25)	18 (18)	
*Gender*			0.957
Male	59 (67)	65 (64)	
Female	29 (33)	34 (33)	
In a relationship	75 (85)	80 (79)	0.276
Has children	66 (75)	68 (72)	0.069
Average number of children	2.1 ± 1.5 (0–6)	(0–5) 1.85 ± 1.5	0.423
Regular physical activity	34 (39)	36 (36)	0.884
Moderate or high alcohol consumption	28 (32)	25 (25)	0.268
Use of non-prescription psychoactive substances	4 (4.5)	2 (2)	0.406
Seniority			0.048
Attending physicians	49 (55.7)	47 (47)	
Resident	39 (44.3)	52 (51)	
Years of practicing medicine			0.522
< 5	40 (45)	31 (31)	
5–15	31 (35)	39 (39)	
≥ 16	17 (20)	30 (29)	
*Year of working in the ED*			0.104
< 5	40 (45)	66 (65)	
5–15	21 (24)	27 (27)	
≥ 16	27 (31)	7 (7)	
*Weekly clinical hours in the ED*			0.001
< 40	25 (28.5)	31 (31)	
40–50	37 (42)	33 (33)	
≥ 51	26 (29.5)	31 (31)	
*Weekly clinical hours not in the ED*			0.059
0	54 (61)	65 (64)	
< 30	26 (30)	28 (28)	
> 30	8 (9)	1 (1)	
*Monthly 24-h shifts*			0.879
0	20 (23)	19 (19)	
1–3	9 (11)	8 (8)	
4–6	48 (54)	60 (59)	
> 7	11 (13)	12 (12)	
*Minutes traveling to work per day*			0.648
< 10	14 (16)	10 (10)	
10–30	34 (39)	32 (32)	
30–60	29 (33)	44 (44)	
> 60	11 (12)	9 (9)	
*Yearly vacation days used*			0.014
0–5	6 (7)	17 (17)	
06–10	24 (27)	36 (36)	
11–20	37 (42)	37 (37)	
> 20	21 (24)	10 (10)	

### Stage 1: emergency physician burnout before the pandemic

Two-hundred and forty questionnaires were sent and 84 out of 88 were completed in full, for a 35% compliance rate. Four participants did not complete this stage and were excluded.

Fifty-eight participants (70%) reported at least one symptom of burnout, and all but one (n = 57, 69% of the total Stage 1 cohort) had a high emotional exhaustion score (see Table 2 –Maslach score (pre-pandemic)). Thirty-one participants (37% of responders) of the burned-out group (37.8% of the cohort) also had a high depersonalization score. Only four participants (5% of the cohort) had a low personal accomplishment score, and they all had high emotional exhaustion and depersonalization scores.

**Table 2:** Maslach Score for the first (pre-pandemic) group (N = 84)

Index	High score	Medium score	Low score	Mean (SD)
Emotional exhaustion, n (%)	**57 (69%)**	15 (18%)	12 (12%)	29 (1.5)
Depersonalization, n (%)	**31 (37%)**	24 (29%)	27 (32%)	10 (0.8)
Personal accomplishment, n (%)	49 (49%)	29 (35%)	**4 (5%)**	40 (0.5)

**Bold** – score indicating high burnout

### Work and meaning inventory (WAMI)

Most participants (n = 69, 78%) had a high WAMI score. The average score of the cohort was 34 (out of a maximal mean score of 50, SD = 6). There was a significant correlation between the burnout index and self-reported depression and hopelessness (r = 0.59, *p* < 0.001). Burnout was significantly and negatively correlated with age (r = − 0.36, *p* < 0.001), engaging in physical activity (r = − 0.31, *p* = 0.004), and number of years working in the ED (r = − 0.43, *p* < 0.001). The burnout index correlated significantly with the number of 24-h shifts (r = 0.33, *p* = 0.002). The number of work hours outside the ED had a negative correlation (r = − 0.24 *p* = 0.025) with the burnout index. There was no significant correlation between burnout and number of children, non-prescription psychoactive substance use, alcohol consumption, number of vacation days, and travel time to the workplace.

### Stage 2: burnout during the pandemic

For this stage, a total of 393 questionnaires were sent of which 101 were returned for a 26% compliance rate. Eight participants did not complete the Maslach and/or WMAI part of the questionnaire and were excluded, thus yielding a cohort of 93 participants. Most of the responders (n = 79, 85%) reported at least one symptom of burnout, all 79 reported having emotional exhaustion and 55 (59%) also had depersonalization. The great majority (n = 85, 91%) had a high personal accomplishment score (See Table 3-Maslach score (pandemic)).

**Table 3:** Maslach score for the second (pandemic) group (N = 93)

Index	High score	Medium score	Low score	Mean (SD)
Emotional exhaustion, n (%)	**79 (85%)**	13 (14%)	1 (1%)	36 (1.8)
Depersonalization, n (%)	**55 (59%)**	26 (31%)	12 (13%)	13 (0.8)
Personal accomplishment, n (%)	85 (91%)	8 (9%)	**0 (0%)**	44 (1.6)

**Bold** – score indicating high burnout

### WAMI score

Most of the participants (n = 85, 91%) had a high (> 35) WAMI score. The average score was 40 out of a maximum score of 50. There was a significant negative correlation between the emotional exhaustion and depersonalization and the WAMI score (*p* < 0.001). Similarly to the findings in the pre-COVID-19 group, burnout and physician age were significantly and negatively correlated (r = -0.22 *p* = 0.015). The correlation between the burnout index and the number of 24-h shifts per week was also significant (r = 0.23, *p* = 0.015). There was no correlation between engaging in physical activity, non-prescription psychoactive substance use, or alcohol consumption and burnout in this group (*p* = 0.241 and *p* = 0.348, respectively).

### Burnout factors unique to the pandemic

Factors found to be especially disturbing (a score > 2.5 out of 3 maximal score) for EPs during the pandemic were fear of infecting family members (2.61, SD 0.61), lack of daycare centers for the participants’ children (2.62, SD 0.74), increased workload (2.58, SD 0.63), and lack of sufficient logistic support (2.55, 0.72) (Table 4).

**Table 4:** Unique pandemic stress factors

Stress Factor	Mean score (range 1–3)	Std deviation	Number of participants stating the factor as being irrelevant for them
Social distancing from loved ones (non-nuclear family)	2.49	0.68	6
Increase in working hours	2.38	0.69	7
Increase in workload	2.58	0.63	1
Shortage in protective gear	2.13	0.85	16
Need to wear protective gear while working	2.48	0.67	1
Exposure to the virus in the workplace	2.25	0.69	1
Fear of infecting family members	2.63	0.61	6
Fear of infecting co-workers	2.51	0.67	4
Need to support family member in the setting of increased workload	2.25	0.76	14
Inability to give adequate medical care	2.31	0.72	7
Lack of sufficient logistic support	2.55	0.72	12
Reduced wages	2.3	0.80	31
No educational or daycare centers for children	2.62	0.74	39^a^

^a^20 had no children

The factor that emerged as being most helpful for the participant's well-being was the support of family and friends (2.69, SD 0.57) (Table 5).

**Table 5:** Supporting factors unique to the pandemic

Supporting factor	Mean score (range 1–3)	Std deviation	Number of participants stating the factor as being irrelevant for them
Family and friends	2.69	0.57	1
Co-workers	2.50	0.66	3
Community	1.99	0.88	9
Management	2.02	0.88	5
Improvement of physical working conditions	2.17	0.88	9
Reduced working hours	2.43	0.84	38
Working 12/24 shifts	1.77	0.90	17^a^
Improved wages	2.43	0.76	26

^a^those who stated that the switch to 12/24-h shifts was helpful had an average of 5.13 24-h shifts per month, compared to an average of 3.8 for those who found the change unhelpful

We ended the questionnaire with an open-ended question requesting the participants to state what they thought might help them better cope with the extra workload caused by the pandemic. The most frequent responses were shortening working hours (12 responders), adding supporting non-medical staff (9 responders), and improving physical work conditions (9 responders).

### Comparing pre- and post-COVID-19 groups

Group matching of the pre-COVID-19 (n = 84) and post-COVID-19 (n = 93) revealed that the burnout index was significantly worse during the pandemic. The Maslach scores for items pertaining to emotional exhaustion and depersonalization were significantly higher in the pandemic group compared to the pre-pandemic group (20% and 28%, *p* = 0.004 and *p* = 0.007, respectively). In contrast, the feeling of personal accomplishment was significantly higher in the pandemic group among whom 91% had a high score compared to only 49% in the pre-pandemic group (*p* = 0.012). The WAMI score was also significantly improved in the pandemic group, with a mean difference of 4.5 points compared to the pre-pandemic group (*p* < 0.001).

Individual matching of the 39 physicians who filled both the pre and post COVID-19 questionnaires demonstrated a similar trend to higher rates of emotional exhaustion and depersonalization, as well as a stronger feeling of professional accomplishment, although the *p* values did not reach a level of significance, probably due to the small sample size. The WAMI score was higher in the COVID-19 questionnaires compared to the pre-pandemic questionnaires (a change of 4.1 points with an SD of 1, *p* < *0.001*). The results of an additional analysis of the participants who completed only one questionnaire showed a similar trend, and they were mostly statistically significant (Table 6).

**Table 6:** Pre-pandemic and pandemic burnout, independent groups

	Mean pre-pandemic (n = 45)	Mean pandemic (n = 54)	*p* value	Mean difference	SD
Emotional exhaustion	33	40	0.008	7	3
Depersonalization	13	14	0.216	1	2
Professional accomplishment	40	47	< 0.001	7	1
WAMI	35	40	0.005	4	2

### Stage 3: intervention

Twenty physicians participated in a workshop intervention, and 13 of them completed all questionnaires (i,e,, both pre-COVID-19 and during COVID-19) immediately following the workshop and three months later, and they were included in a further analysis. They all reported a significant increase in burnout during the COVID-19 pandemic. Following the workshop, 8 physicians reported a lessening of the burnout levels and a mild improvement in work meaning scores, but these positive changes were not sustained at the 3-month interval (for example, the WAMI scores in Table 7).

**Table 7:** WAMI scores for the 14 workshop participants

Participant	Before COVID-19	During COVID-19	Immediately after the workshop	3 months after the workshop
1	40	41	46	39
2	40	37	37	27
3	39	44	45	38
4	41	49	50	50
5	36	36	33	37
6	31	39	35	
7	41	48	42	42
8	41	38	44	41
9	38	23	38	27
10	41	40	41	43
11	25	30	17	28
12	36	32	33	35
13	37	32	25	29
14	39	42	44	39

## Discussion

Physician burnout is a major global problem and an even greater challenge during the current pandemic. [19] The literature points to the disturbing fact that over one-half of resident physicians suffer from burnout, and that over one-quarter of them show symptoms of depression. [20–23].

Several Israeli studies investigated the prevalence of burnout among resident and attending physicians. Their results showed high levels of burnout (similar to those shown in international studies), as well as a significant increase in the rate of this phenomenon over the years. [24].

Both pre-pandemic and pandemic groups had a high percentage of moderate or high self-reported alcohol consumption (32% and 25%, respectively), relative to Israel’s general population which reportedly has one of lowest alcohol consumption rates per-capita in the OECD countries. [25]. To the best of our knowledge, there are no data regarding alcohol consumption among Israeli physicians. Statistics from other countries show that alcohol consumption among physician is not uncommon, and that hazardous alcohol consumption (not evaluated in our study) was also not rare (ranging from 12 to 25% in different studies) [26, 27]. Some studies found an association between alcohol consumption and burnout, which was not found in our study. [28, 29].

### Burnout among EPs

EPs worldwide appear to suffer a higher rate of burnout compared to other specialties. [1, 6–9] Paradoxically, they also have high rates of work satisfaction [8, 10, 11]. The unique characteristics of the ED work settings may be a contributing factor. In Israel, EPs are most likely to leave the profession compared to all other fields [30, 31] These findings are compatible with the high level of burnout both before and during the pandemic found in the present study. A 2015 study conducted in Israeli EDs found two potential aspects of a physician-specific (rather than organizational-specific) intervention to prevent burnout: they were decreased work-related worry and an increased sense of existential meaning derived from work. [10].

The current study findings regarding the contributing and protective factors correlate well with those shown in previous studies on physician burnout. The factors that contributed to the high levels of burnout in previous studies included high workload, sleep deprivation, patient complaints and the associated fear of lawsuits, loneliness, handling “difficult” patients, and the lack of appropriate monetary compensation [11], as well as administrative chores, and difficulties with work-life balance [30]. Work variety and interest, as well as care for the physicians’ own health were suggested to be factors that protected from burnout [30]. Younger physicians were reportedly more prone to burnout. [19]. We, as did others [19] also found that high numbers of weekly hours and of 24-h shifts correlated to a high burnout index, and that younger physicians are more likely to sustain burnout symptoms,

### Burnout during a pandemic

Definitive data on this contemporary topic are limited, but some interesting trends have been shown in the studies published thus far. Shanafelt et al. conducted interviews on causes of concern regarding the pandemic among a group of 69 staff members during the first week of the COVID-19 pandemic. Those authors found that lack of protective equipment, exposure to the virus in the workplace, the risk of infecting family members, and the need to provide care not in their field of expertise were the main causes of concern at that time [14]. A literature review of articles on the impact of previous pandemics and natural disasters on the mental health of healthcare workers concluded that these events increase burnout and may cause post-traumatic stress disorder. [12]. Another recent review concluded that EPs as well as public health, primary care, and intensive or critical care workers are particularly at high risk of developing psychological symptoms due to the COVID-19 pandemic. [13].

The present study showed an expected increase in emotional exhaustion as well as depersonalization, in parallel with an increase in feeling of professional accomplishment that may serve to protect against burnout, as seen in their WAMI scores. A Canadian study that was carried out after the first ten weeks of the pandemic found that personal safety, academic and educational work, personal protective equipment, the workforce, patient volumes, work patterns, and work environment had an impact on the well-being of EPs, and that burnout levels remained stable [32]. Another finding was that the lack of daycare and educational centers for the physicians’ children during quarantine was a significant burnout factor. Importantly, this is a preventable cause for burnout.

During the first wave of the COVID-19 pandemic, ED staff members started working in “capsules” consisting of permanent staffs of nurses and doctors working 12-h shifts with 24-h breaks in-between in order to reduce the risk of isolation of an entire team in case of exposure. Opinion varied regarding the usefulness of this method in preventing burnout. Those who favored it were the ones who had an average of 5.13 24-h shift per month compared to the ones who opposed it who had an average of 3.8 24-h shifts per month.

### Interventions to reduce burnout

A meta-analysis of over 50 studies that focused on physician burnout described two major strategies of burnout interventions: one was individual-focused and the other was structural/organizational-focused. According to its findings, clinically meaningful reductions in burnout among physicians can be achieved using both strategies, although long-lasting effects were not evaluated in depth in those reports. [33] Our study found limited benefit to an individual-focused resilience-based intervention in reducing EM physicians’ burnout during the pandemic. We can only speculate about the reasons for this limited benefit. Possible reasons may include, but are not limited to, the short duration of the intervention compared to long years of burnout, the failure to address the issue of personal and familial COVID-19 illness during workshops, and the lack of concurrent organizational intervention.

### Study limitations

We analyzed our study cohort by means of group matching, and some individuals would therefore inherently be included in both groups. Due to COVID-19 restrictions, the intervention was performed in only one medical center, and the relatively small numbers of participants may not represent all Israeli EPs. The pandemic group had a higher ratio of medical and surgical residents who had fewer years working in the ED and more 24-h shifts, two factors that correlate with high burnout.

## Conclusions

Burnout is a major issue for EPs and even more so during a pandemic. However, being in the frontlines of this worldwide struggle has led to an increase in the appreciation of the value of their work on the part of the physicians. Some of the major pandemic specific burnout factors are preventable and should be considered in work plans for dealing with future outbreaks.

We recommend combining continuous individual-focused burnout reduction intervention with organizational-focused intervention, during routine and crisis times. We also recommend decision makers to reach out to EPs, to better understand their need in real-time, and enable proper resource allocation for useful burnout reduction.

Further research is warranted to determine which factors will be most helpful in preventing burnout among EPs, as well as the lasting effects of the pandemic on their well-being.

## Data Availability

The datasets used and/or analyzed during the current study are available from the corresponding author on reasonable request.

## Abbreviations

EM: Emergency medicine
ED: Emergency department, COVID-19-coronavirus disease of 2019
WAMI: Work and meaning inventory

## Footnotes

1 Part of the goal to devise a national intervention plan to reduce EM physician burnout.

2 The Tel Aviv Medical Center. The participation of other medical centers was not possible due to COVID-19 restrictions that were in place at the time.
